# A Temporal Network Based on Characterizing and Extracting Time Series in Copper Smelting for Predicting Matte Grade

**DOI:** 10.3390/s24237492

**Published:** 2024-11-24

**Authors:** Junjia Zhang, Zhuorui Li, Enzhi Wang, Bin Yu, Jiangping Li, Jun Ma

**Affiliations:** 1 Faculty of Information Engineering and Automation, Kunming University of Science and Technology, Kunming 650500, China; zhangjunjia@stu.kust.edu.cn (J.Z.); mjun@kust.edu.cn (J.M.); 2 Yunnan Key Laboratory of Intelligent Control and Application, Kunming University of Science and Technology, Kunming 650500, China; 3 Chuxiong Dianzhong Nonferrous Metals Co., Ltd., Chuxiong 675000, China; 15125721994@163.com (E.W.); ybsx2024@126.com (B.Y.); wy50929170pinghe@163.com (J.L.)

**Keywords:** copper smelting process sensors’ data, temporal representation and feature extraction, temporal convolutional network, matte grade prediction

## Abstract

Addressing the issues of low prediction accuracy and poor interpretability in traditional matte grade prediction models, which rely on pre-smelting input and assay data for regression, we incorporate process sensors’ data and propose a temporal network based on Time to Vector (Time2Vec) and temporal convolutional network combined with temporal multi-head attention (TCN-TMHA) to tackle the weak temporal characteristics and uncertain periodic information in the copper smelting process. Firstly, we employed the maximum information coefficient (MIC) criterion to select temporal process sensors’ data strongly correlated with matte grade. Secondly, we used a Time2Vec module to extract periodic information from the copper smelting process variables, incorporates time series processing directly into the prediction model. Finally, we implemented the TCN-TMHA module and used specific weighting mechanisms to assign weights to the input features and prioritize relevant key time step features. Experimental results indicate that the proposed model yields more accurate predictions of copper content, and the coefficient of determination (*R*^2^) is improved by 2.13% to 11.95% and reduced compared to the existing matte grade prediction models.

## 1. Introduction

In the copper smelting process, the copper matte grade is an important production indicator that reflects the quality of the smelting process. A suitable matte grade can reduce the material consumption and blowing time in the subsequent converter smelting, while an unsuitable grade will increase consumption [1]. The stability of the matte grade is essential for ensuring the efficient and safe operation of subsequent production [2]. However, the manual sampling, cooling, and analysis of matte are conducted during the smelting process, resulting in a time delay of over one hour. Due to this detection delay and human factors, the measured matte grade indicators are difficult to feedback in a timely manner to the furnace system, which hinders the tuning of the control system [3]. Therefore, how to accurately predict the matte grade to optimize the operating parameters and improve the efficiency of copper smelting process has been a challenge for managers and researchers [4].

Currently, data-driven based copper grade prediction methods include regression prediction and recursive prediction. Regression prediction methods construct linear mathematical models by analyzing the relationships between variables, characterized by high accuracy and ease of implementation. Since 2007, these methods have been widely used in copper grade prediction [2]. Weihua Gui et al. [2] developed an early copper grade prediction model using multi-phase fuzzy neural network with constrained gradient descent for parameter updating. Shouyi Yu et al. [5] and Luo Zhao et al. [6] proposed a method based on back propagation neural networks (BP) to mine the parameter information among copper grade, copper temperature, and the silicon–iron ratio in slag to achieve copper grade prediction. Peng Deng et al. [7] combined long short-term memory networks (LSTMs) with mechanistic models to predict four parameters (oxygen enrichment, abrasive grade, control parameters, and temperature). Xincai Li et al. [8] optimized support vector machines (SVMs) using sparrow search algorithm to establish a nonlinear relationship model between copper grade and the amounts of copper ore, fuel, and ore composition for prediction. Peng Xiaobo et al. [9] considered the difficulty of statistically and mechanistically determining the parameters. They proposed a dynamic T-S recursive fuzzy neural network to derive the influence weights of various mineral quantities and constructed a soft measurement model for copper grade. Although these regression-based prediction methods achieved satisfying results, they rely on real-time process variable inputs for output results, and this leads to less interpretability for intermediate processes and low accuracy of prediction results. This still fails to address the issues of guidance lag in production and the timely feedback needed for furnace parameter adjustments.

In recent years, with the growing demand and development of industrial prediction, recursive prediction based on deep learning has gradually become a trend, addressing the aforementioned issues [10]. This method predicts the copper grade of the next furnace by learning patterns from historical sensor process data to give on-site personnel enough time and strategies to adjust and guide production. Current methods include artificial neural networks (ANNs) [11], temporal convolutional networks (TCNs) [12], bidirectional long short-term memory networks (BiLSTMs) [13], BiGRU [14], and the Transformer network proposed by the Google team [15] and its variant, Informer [16]. Among these, the TCN model uses residual connections with convolution to control the feature extraction process, making it more effective than convolutional neural networks (CNNs) in handling sequential data with time characteristics, and allowing it to recursively capture temporal patterns, thus becoming a popular model for time series prediction. Mao et al. [10] proposed a recursive model that takes into account the temporal analysis in the field of matte grade prediction and demonstrated that, by considering temporal correlations, the model yields more accurate predictions. Lin et al. [17] applied the TCN to perform multi-layer convolution operations on the input time feature sequences, enabling the learning of global temporal feature information. Liang et al. [18] utilized stacked TCNs with attention mechanisms for spatiotemporal prediction. It is shown that leveraging the model can effectively enhance prediction accuracy. Pan et al. [19] performed sequential prediction through the extraction of multi-period features under different time windows.

The TCN model with its powerful residual structure and dilated causal convolution pattern, effectively captures temporal information within large-scale data, making it a popular choice for time series prediction [20]. However, as noted in the aforementioned [17,18,19], although TCN can capture dependencies within a certain time range, its hierarchical structure limits the growth of the receptive field as the depth increases. This limitation makes it difficult for TCN to cover the global information of long time series, preventing it from fully capturing critical temporal information. Moreover, with the advent of the Transformer module and the attention mechanism [15], there has been a significant advantage in capturing long-term dependencies and global information, which enables better extraction of critical temporal features. Consequently, attention mechanisms are commonly incorporated into the current time series prediction to achieve higher prediction accuracy. Guangxun E et al. [21] introduced attention mechanisms in 1D-CNN to explore the temporal and spatial correlations of multi-dimensional feature sequences for high-precision predictions. Li et al. [22] introduced a dual attention mechanism for feature space and temporal aspects based on TCN, dynamically obtaining important metrics from both feature and time dimensions to improve prediction accuracy. Yang et al. [23] integrated a multi-head attention into the traditional generative adversarial network (GAN) method to better learn the latent structure of chillers’ fault data. Li et al. [24] utilized the improved layer-wise relevance propagation (ImLRP) method, employing attention relevance scores to assess the contribution of each neuron to the output. Zhao et al. [25] proposed a convolutional adversarial multi-wavelet neural network (CAMCNN) to address the issue of insufficient utilization of target data for feature extraction. The prediction accuracy of TCN, LSTM, and GRU models was improved by incorporating an attention mechanism. It is evident that the attention mechanism can effectively help the model focus on the most important parts of the input data, thereby enhancing its ability to capture critical information. This mechanism allows the model to dynamically adjust its attention to different time steps in the input sequence based on the current task, making it more flexible and efficient when processing complex time series data.

However, considering the inconsistent production periods in copper smelting process, this leads to the following issues:

(1) Uncertain periodic information of input variables: Due to the influence of actual industrial production on-site, there may be irregular production intervals caused by human factors. With fluctuating amounts added, the periodic characteristics of the time series information are unclear.
(2) Difficult to extract periodic features: In the copper smelting process, the influence of temporal characteristics for different input variables on the output results is different at each time step. It is essential to enhance the weights of input variables at key time steps, which can extract the key periodic features to ensure the accuracy of matte grade prediction.

In response to the abovementioned issues, many scholars in the industrial field have adopted the following solutions: Fargalla Mandella et al. [26] proposed a combination model called Time2Vec attention-based CNN-BiGRU neural network, Zhou et al. [27] introduced a model called an adaptive timing encoding mechanism based Transformer with Time2Vec, Li et al. [28] developed the PSO-TCN-attention neural network, and Cai et al. [29] presented a malicious network traffic detection model based on a bidirectional temporal convolutional network with a multi-head self-attention mechanism. All these models have effectively addressed various time-related issues in their respective fields, achieving excellent results. Therefore, we build upon their research and propose improvements, proposing a novel matte grade prediction method based on Time2Vec and TCN-TMHA, and focusing on enhancing the temporal periodicity of the input variables and extracting key time step features within the production cycle. The main contributions and innovations of this paper are as follows:

(a) Utilization of Time to Vector (Time2Vec) for temporal embedding: The Time2Vec temporal embedding layer captures periodic relationships among different copper smelting monitoring data variables, thereby constructing periodic characteristics. This provides a supportive approach to build periodic features that are fed into the predictive model, offering robust data support for subsequent matte grade prediction.
(b) Combination Model of TCN-Temporal Multi-Head Attention (TMHA): A combination model of TCN-TMHA is utilized. This model leverages dilated causal convolutions and residual connections to enhance the modeling of complex temporal dependencies in the copper smelting process. It also employs TMHA mechanisms to explore the coupling relationships between process variables and copper grade, while using specific weighting mechanisms to enhance the representation of time steps and input features, thereby strengthening the information expression of important periodic features.
(c) Addressing Dependency on Real-Time Process Variables: In the field of copper grade prediction, this method resolves the issue of regression prediction relying on real-time process variable inputs. By using recursive models for time series prediction that do not depend on real-time data, it leverages historical data to anticipate future copper grade levels, enabling timely on-site parameter adjustments and improving prediction accuracy.

## 2. Theory and Methodology

### 2.1. Problem Description

Our datasets are based on production data from a copper smelting enterprise in the southwestern region of China.

**Figure 1:**
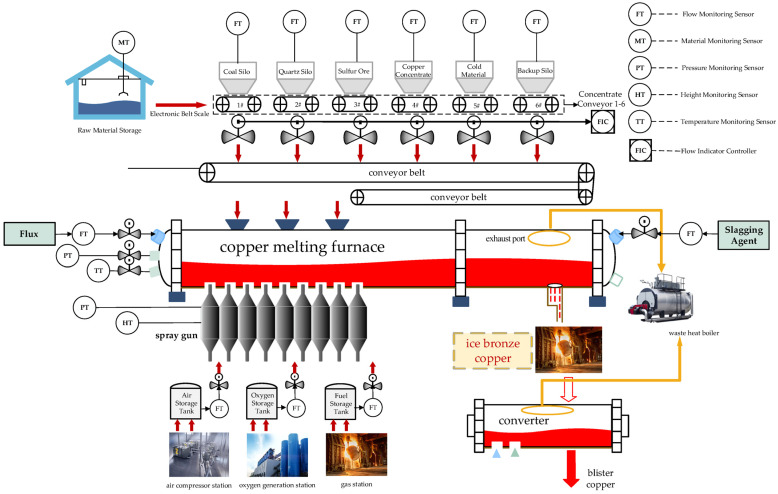
The process variables collected by sensors for matte grade measurement.

As shown in Figure 1, the copper smelting process aims to obtain blister copper. First, ore is obtained from the raw material storage, and then, materials coal, quartz, sulfur ore, copper concentrate and cold material are added and mixed before being fed into the copper smelting furnace. To achieve the desired grade of blister copper, it is necessary to adjust the opening of the burner, fuel, and flux using sensors and control valves to regulate the production process. However, the grade of blister copper cannot be measured directly by sensors, so data from other sensors are used for prediction.

Traditional regression prediction methods require the construction of a soft measurement regression model, which cannot provide timely guidance for production. Therefore, this paper employs a time series recursive model to explore the temporal patterns of data changes in the copper smelting process, thus enabling the prediction of blister copper grade based on historical time steps.

We establish a prediction model for the grade of blister copper and initially select 19 process data inputs obtained through sensor collection and manual analysis as time series inputs. These inputs include the contents of Cu, S, Fe, Si, and Ca in the copper concentrate, the amount of flux added, the iron-silicon ratio in the slag, oxygen concentration, oxygen flow rate, molten pool temperature, and burner height, represented as time series inputs *x*1~*x*19, the target label for predicting copper grade is denoted as *Y*.

In the copper smelting process, where the time cycle is not fixed (ranging from 55 to 70 min), various input time series data exhibit inter-coupling, weak periodic expressions, and a lack of distinct time step characteristics. These issues bring challenges for sequential prediction of matte grade. Therefore, the focus of this study is to enhance the periodicity of the input data and increase the weight of the input features at key time steps in the sequence, thereby achieving a relatively high accuracy in the sequential prediction of matte grade.

#### 2.1.1. Characterization of the Periodicity of Time Series Signals

**Figure 2:**
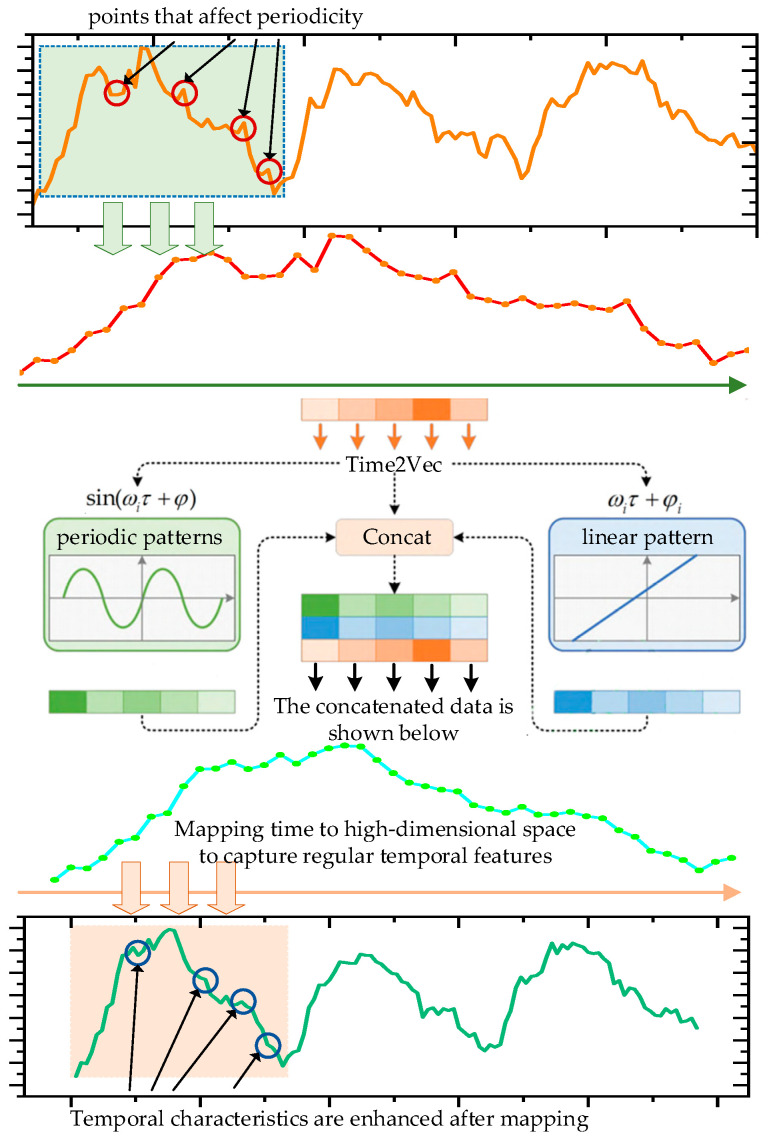
Periodic representation of time series signals based on Time2Vec.

As an example, the original time series characteristics of the copper concentrate input are shown in Figure 2 (upper). Due to the influence of actual industrial production on-site, the production cycle is irregular, and anomalies caused by human factors result in the periodicity of the time series data being unclear. How to represent the periodic and non-periodic characteristics of the original data, thereby showcasing the time series characteristics of the production data, is one of the challenges faced in predicting the grade of blister copper.

To address this, we utilize Time2Vec to encode the input time series data, providing a representation of temporal information across multiple time series [23]. The structure of Time2Vec is illustrated in Figure 2.

Convert time data into representations for both periodic and non-periodic features, which allows for the extraction of high-order feature representations as follows:

(1) $$Time2vec(\tau )\left[i\right]=\left\{\begin{array}{l} \omega_{i}\tau +\varphi_{i}\;if\;i=0\\ \sin(\omega_{i}\tau +\varphi )\;if\;0<i<k \end{array}\right.$$

where *k* is the variable that defines the periodic regions within the high-order features. For the *i* component, when *i* = 0, it represents a linear component, while for 0<i<k, it indicates periodicity, and $\sin(\omega_{i}\tau +\varphi )$ is used to capture the periodic patterns of time. Additionally, $\omega_{i}\tau +\varphi_{i}$ captures the non-periodic patterns of time. By combining Time2Vec module with time series signal preprocessing, it assists the model in extracting key temporal prediction features from the time series data.

#### 2.1.2. Periodic Feature Extraction Based on TMHA

The TMHA mechanism calculates weights to determine the importance of features obtained from various sensors during the copper smelting process. Different sequence segments within the process variable grouping can represent diverse information such as temperature, pressure, and material input quantity. The multi-head self-attention mechanism captures this diversity in input information and aggregates it, enhancing the model’s ability to capture copper smelting process information.

**Figure 3:**
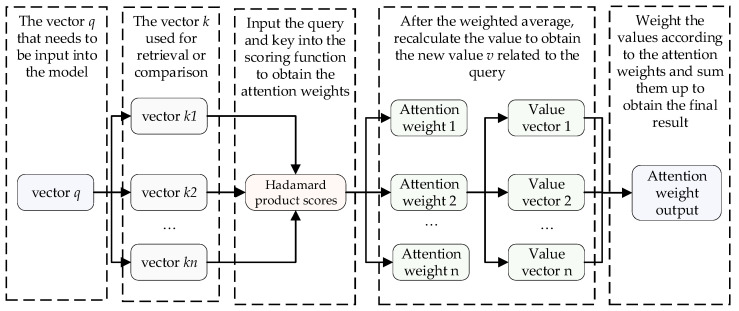
The construction diagram of attention mechanism.

Additionally, due to the presence of noise and outliers in industrial field data, the TMHA mechanism leverages its strong robustness to automatically adjust the weights of these noise values and outliers in classification tasks, thereby reducing their impact on the model and further improving the prediction efficiency of matte grade. The specific steps of the multi-head self-attention mechanism are outlined below, and its general process is illustrated in Figure 3.

We employ TMHA of the temporal Transformer model for feature extraction, providing a weighted representation of each variable at different time steps, which establishes connections and influences between the input data across various time intervals. The length of the original data sequence is *x*, and the dimensionality of the variables is *d*. The input sequence is first linearly mapped into three different matrices *W* to transform it into the query matrix *q*, key matrix *k*, and value matrix *v*, as shown in Equation (2):

(2) $$\begin{array}{l} q=W_{q}\times x\\ k=W_{k}\times x\\ v=W_{v}\times x \end{array}$$

where $W_{q}$, $W_{k}$, and $W_{v}$ is parameter matrices. used to transform the input original data sequence vector *x* into a query matrix *q*, key matrix *k*, and value matrix *v*.

The attention function includes the scaled dot product of the query with all keys *k*, divided by the scaling factor $\sqrt{d_{k}}$. The output matrix is as follows:

(3) $$H=Attention(q,k,v)=soft\max(\frac{qk^{T}}{\sqrt{d_{k}}})$$

According to Equations (4) and (5), this is achieved by using different linear projections to project the queries, keys, and values multiple times.

(4) $$MultiHead(q,k,v)=W^{O}Concat(head_{1},\cdots ,head_{h})$$

(5) $$head_{i}=Attention(W_{i}^{q}q,W_{i}^{k}k,W_{i}^{v}v)$$

where $head_{i}$ represents the results of the self-attention computation for each head, *O* is the output of the multi-head self-attention mechanism, *i* is the number of heads in the multi-head self-attention mechanism, and $W^{O}$ is the parameter matrix.

**Figure 4:**
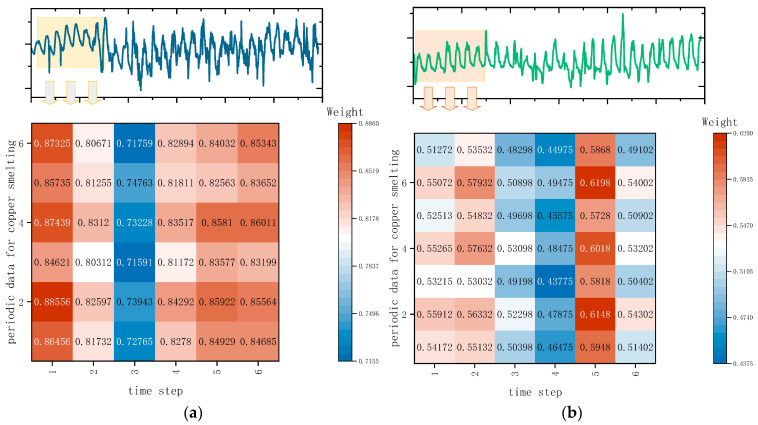
Weighting time steps of sensor process data based on attention mechanism. (**a**) Example 1: weight map of copper concentrate data over 6 cycles and 6 time steps. (**b**) Example 2: weight map of oxygen enrichment concentration data over 6 time steps.

As shown in Figure 4, the original time series data have been assigned attention weights over six cycles. Following the steps described earlier, we can obtain the weights of various inputs at different time steps, thereby enabling feature extraction for critical time steps. Taking copper concentrate feed data (left) and oxygen enrichment concentration (right) as examples, the attention mechanism computes the weights for each variable across the six time steps (one cycle), allowing for the extraction of features from critical time steps. In terms of the attention map for oxygen enrichment concentration, it can be observed that for the six sets of data, the first, second, and fifth time steps of the smelting process (0–10 min, 10–20 min, and 40–50 min) have a greater impact.

### 2.2. The Benchmark Model TCN for Time Series Prediction

#### 2.2.1. Time Series Prediction Pattern Framework

**Figure 5:**
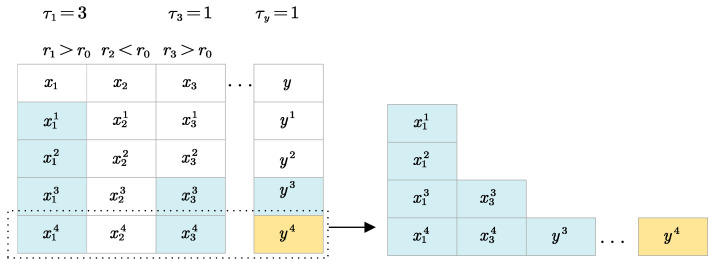
Description of real-time prediction issues in regression prediction.

To illustrate our time series forecasting methods, we compare time series models that rely on historical data with regression models that depend on real-time data; as shown in Figure 5.In regression prediction, we typically seek a nonlinear model relationship for *x*1, *x*2, …, *xn* = *y*, which is constructed with high accuracy and reliability. However, this model necessitates real-time input data *x* to obtain *y*. On the other hand, the current temporal recurrent model seeks y at the present moment by considering the entire historical data of *x* and *y* time steps, thereby possessing the characteristic of not relying on real-time data and solving issues related to real-time data.

**Figure 6:**
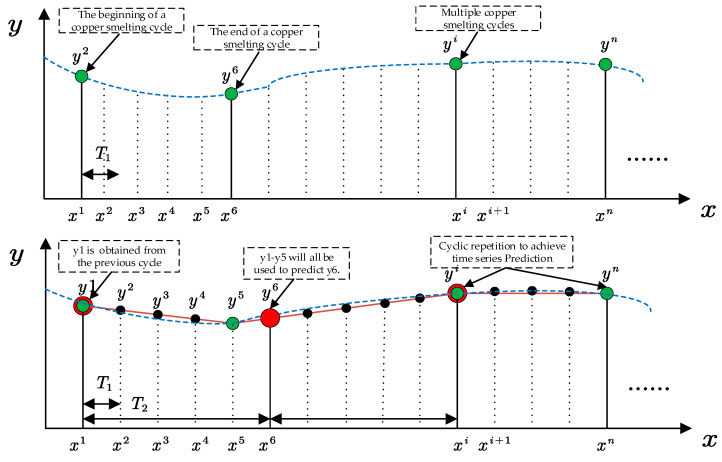
Copper smelting process time cycle.

As shown in Figure 6, the key temporal step feature highlighted in our article is represented by each *T*1 denoted by *x*1-*x*2. Multiple *T*1 combined constitute a complete copper smelting process *T*2. The temporal step feature we need to extract is the temporal step weights of each *T*1 within one *T*2. This serves two purposes: one is to weigh the time steps to improve prediction accuracy, and the other is to identify the time steps influencing the smelting cycle, allowing on-site workers to pay more attention to these moments, thereby enhancing safety.

The dilated causal convolution in the TCN model effectively captures temporal dependencies in time series data, relying on information from previous time steps. Meanwhile, residual connections assist the model in learning identity mappings to preserve critical features from the original input. Together, these components enable TCN to accurately extract and model complex temporal step features.

#### 2.2.2. TCN Feature Convolution Extraction

**Figure 7:**
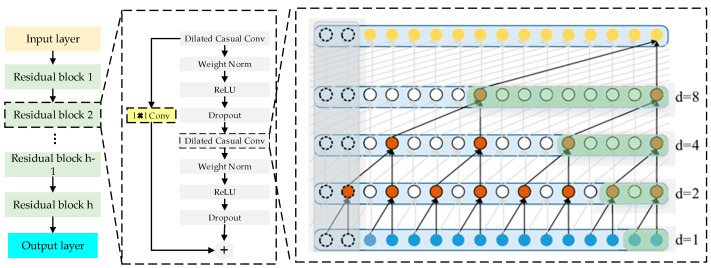
The construction diagram of TCN.

The TCN with causal convolution and residual connections is shown in Figure 7. The calculation formula for the dilated causal convolution is as follows:

(6) $$F(t)=\sum_{i=0}^{K-1}f(i)x_{T-di}$$

where f(i) represents the function of input data where the output of causal convolution at each time step depends on previous time steps. $x_{T-di}$ is for obtaining the corresponding input values. It refers to the distance value corresponding from time *T* to the past time *t*.

The formula for the changed receptive field size *y* is as follow:

(7) $$y=\sum_{n-1}^{n}\left(k-1\right)*d_{n}+1$$

where *n* denote the number of hidden layers, *k* denotes the size of the convolution kernel, and $d_{n}$ denote the dilation factor of the *n*-th hidden layer.

(8) $$X_{h}=\delta (G(X_{h-1})+X_{h-1})$$

where δ(·) represents the transformation, and $G(X_{h-1})$ fuses dilated convolution, weight normalization, activation function, and dropout, which are four components. $X_{h-1}$ denotes the output *h* − 1 of the residual block, while $X_{h}$ denotes the output *h* of the current residual block.

## 3. Framework and Methods for Predicting Matte Grade

**Figure 8:**
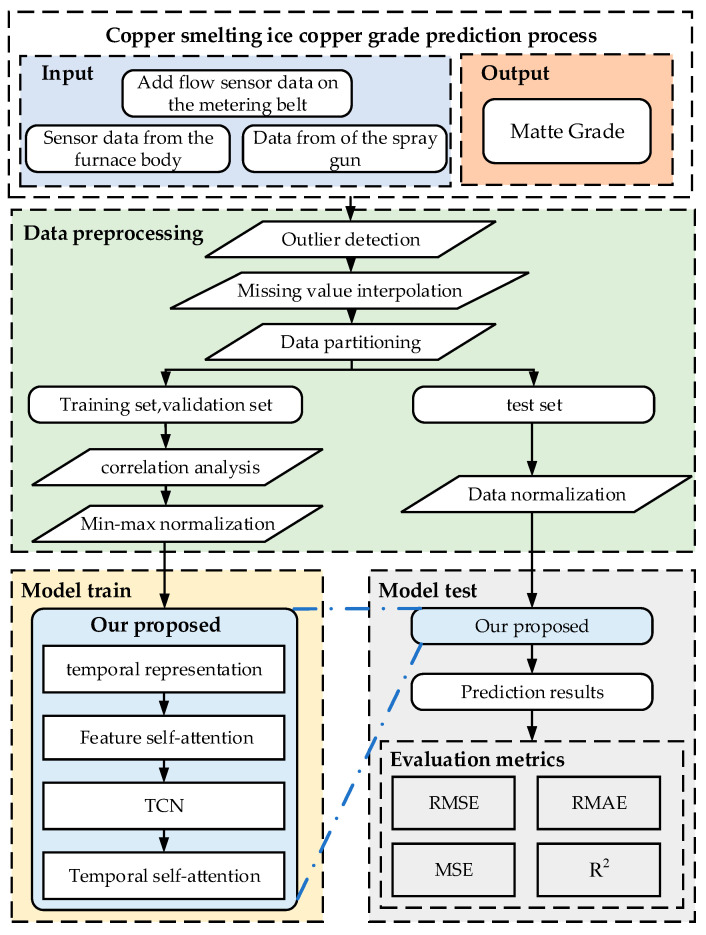
Flowchart of the copper matte grade prediction method.

The steps for predicting matte grade are illustrated in Figure 8. The prediction is achieved through three phases: data preprocessing and feature selection, model training, and model testing. The specific steps are as follows:

Step 1: Input the raw dataset (including ore composition data, addition amount data, and real-time production data) into the MIC-based copper smelting data preprocessing module for feature variable selection, missing value imputation, and normalization.

Step 2: Input the preprocessed data into the Time2Vec and TCN-TMHA training models to complete model training. Time2Vec enhances the temporal periodicity of the input using its data representation method, while the residual connections and attention mechanism of TCN-TMHA elevate the weights of key temporal step features, thereby enhancing the relevant information of input features at key temporal steps. The model is thoroughly trained, and the final output is passed to a fully connected layer to obtain prediction result.

Step 3: Train, validate, and test the model using a 7:2:1 data split. The error correction and comprehensive validation of the proposed model are achieved by using rigorous evaluation metrics, such as *RMSE*, *MAE*, *MAPE*, and *R*^2^, followed by a comparative assessment to verify its effectiveness.

In summary, the core modules of the matte grade prediction model include the maximum information coefficient (MIC)-based copper smelting data preprocessing module, the Time2Vec-based data periodicity representation module, and the TCN-TMHA module for extracting and predicting time-related features in copper smelting production. The following sections will detail these three core modules.

### 3.1. Copper Smelting Data Preprocessing Module Based on MIC

To filter out key sensor process data that affect the copper concentrate grade, quantify the degree of correlation, and reduce the computational resource, MIC is used as the correlation analysis method. Strongly correlated features are selected as inputs for the prediction model. The calculation formula for MIC is as follows:

(9) $$MI(x,y)=\iint p(x,y)\log_{2}\frac{p(x,y)}{p(x)p(y)}dxdy$$

(10) $$MI(D,x,y)=\underset{G\in \Omega }{\max}MI(D/G)$$

(11) $$MIC(x,y)=\underset{mn<B}{\max}\frac{MI(x,y)}{\log_{2}\min\left\{m,n\right\}}$$

where p(x,y) is the joint probability density function of *x* and *y*, and p(x) and p(y) are the marginal density functions. *x* and *y* are, respectively, divided into segments *m* and *n*, forming a *m* × *n* grid *G*, and *B* is the upper bound of the number of divisions.

Due to uncontrollable factors such as equipment maintenance, power outages, oil replenishment, and human factors, there are single-point missing values in the input time series data for material addition amounts, and continuous missing values for monitoring variables like oxygen concentration and molten pool temperature directly affect the temporal continuity of the input data. Therefore, for single-point missing values, the average of the data from the previous and next time points is used to replace the missing data at that time point. For continuous missing values, the continuous values from the two nearest non-missing historical time points are used as a replacement.

Meanwhile, to avoid the influence of differences in parameter dimensions and units on the training effect of the model, the filtered strongly correlated features are subjected to min–max normalization, using Equation (12) to normalize the data.

(12) $$x^{l}=\frac{x_{i}-x_{\min}}{x_{\max}-x_{\min}}$$

where $x^{l}$, $x_{i}$, $x_{\max}$, and $x_{\min}$ represent the normalized value, actual value, maximum value, and minimum value, respectively.

### 3.2. Periodic Time Series Representation Module Based on Time2Vec

The Time2Vec representation results are mapped to a *d*-dimensional space to obtain $Y_{\mathrm{concat}}$, as shown in Equation (13).

(13) $$Y_{\mathrm{concat}}=C_{\mathrm{concat}}(Y,Time2vec(Y))$$

Positional encoding is used to add positional information to the sequence, preserving the temporal characteristics of the original copper smelting data sequence for subsequent modeling. The calculation method for positional encoding is as follows.

(14) $$\left\{\begin{array}{l} E_{pos(t,2d)}=\sin(t/10000^{2d/D})\\ E_{pos(t,2d+1)}=\cos(t/10000^{2d/D}) \end{array}\right.\;for\;d=0,1,2,\cdots ,D/2d-1$$

where $E_{pos(t,2d)}$ and $E_{pos(t,2d+1)}$ presented are the positional encoding calculation for the position embedding at time *t*, and cosine functions are used for odd dimensions, and sine functions are used for even dimensions. The sequence mapped to the *d*-dimensional space is added to the positional encoding results to obtain the sequence input $Y_{\mathrm{concat}}$ with temporal information for the subsequent prediction model.

Finally, the output is integrated with the temporal positional embedding as shown in Equation (15), and the temporal embedding is completed through a linear function.

(15) $$outputs(Z)=Linear(E_{Pos}+Time2vec)$$

### 3.3. Feature Extraction and Prediction for Copper Smelting Production Using TCN-TMHA

**Figure 9:**
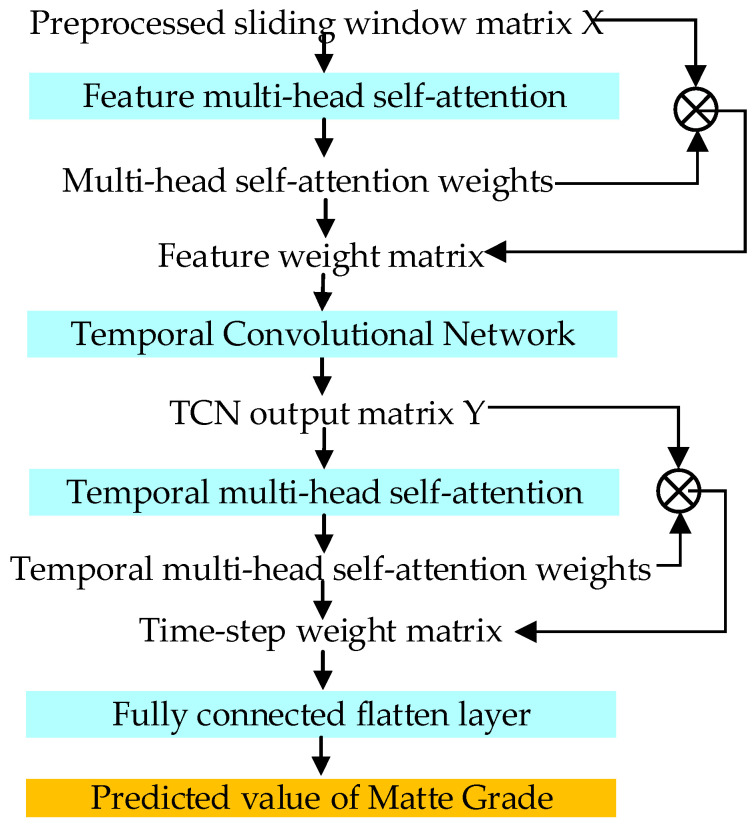
Construction of the TCN-TMHA model.

The data processed by the temporal representation module are inputted into the feature multi-head self-attention layer through the feature matrix *X*. The output quantifies the relationship between the copper content in the molten copper process and the process variable features through the attention weights, as shown in Figure 9.

The TCN-MHTA model demonstrates stronger advantages compared to other time series prediction models such as LSTM and traditional CNNs. TCN effectively captures local and long-range temporal dependencies using dilated causal convolutions, avoiding gradient vanishing and ensuring higher computational efficiency. Meanwhile, MHTA captures multi-dimensional features in the input data through multiple attention heads, flexibly integrating information across long time spans. This combination significantly enhances prediction accuracy when dealing with complex, nonlinear time series data, particularly in tasks like matte grade prediction.

Specifically, weights are assigned to input features of different dimensions using Equation (5) to obtain the feature-weighted matrix, and the calculation process is shown in Equations (16) and (17).

(16) $$\alpha_{n}=MutilHead(q_{1,}k_{1},v_{1})=\left|a_{1},a_{2},\cdots ,a_{k}\right|$$

(17) $$X^{\prime }=\left[\begin{array}{cccc} a_{1}x_{11} & a_{1}x_{12} & \cdots & a_{1}x_{1n}\\ a_{2}x_{21} & a_{2}x_{22} & \cdots & a_{2}x_{2n}\\ \vdots & \vdots & & \vdots \\ a_{k}x_{k1} & a_{k}x_{k2} & \cdots & a_{k}x_{kn} \end{array}\right]$$

Using Equation (8), the feature-weighted matrix $X^{\prime }$ is inputted into the TCN layer. Through the residual block calculation, the output matrix $X^{\prime \prime }$ is obtained; the calculation process is shown in Equations (18) and (19).

(18) $$X^{\prime \prime }=\delta (G(X^{\prime })+X^{\prime })$$

(19) $$X^{\prime \prime }=\left[\begin{array}{cccc} x_{11}^{\prime } & x_{12}^{\prime } & \cdots & x_{1n}^{\prime }\\ x_{21}^{\prime } & x_{22}^{\prime } & \cdots & x_{2n}^{\prime }\\ \vdots & \vdots & & \vdots \\ x_{k1}^{\prime } & x_{k2}^{\prime } & \cdots & x_{kn}^{\prime } \end{array}\right]$$

Then, the output matrix $X^{\prime \prime }$ from the TCN layer is inputted into the temporal multi-head self-attention layer, where weights are assigned for different time steps to obtain the time step-weighted matrix $X^{\prime \prime \prime }$, and this process is shown in Equations (20) and (21).

(20) $$\lambda_{n}=MutilHead(q_{2},k_{2},v_{2})=\left|\lambda_{1},\lambda_{2},\cdots ,\lambda_{k}\right|$$

(21) $$X^{\prime \prime \prime }=\left[\begin{array}{cccc} \lambda_{1}x_{11}^{\prime } & \lambda_{1}x_{12}^{\prime } & \cdots & \lambda_{1}x_{1n}^{\prime }\\ \lambda_{2}x_{21} & a_{2}x_{22} & \cdots & a_{2}x_{2n}\\ \vdots & \vdots & & \vdots \\ a_{k}x_{k1} & a_{k}x_{k2} & \cdots & a_{k}x_{kn} \end{array}\right]$$

Finally, the multi-dimensional time step-weighted matrix $X^{\prime \prime \prime }$ is compressed into a one-dimensional matrix $X^{\left[4\right]}$ through a flattening layer, as shown in Equation (22). The information from the above layers is then integrated through a fully connected layer to output the copper grade prediction value *Y* as shown in Equation (23).

(22) $$X^{\left[4\right]}=\left[\lambda_{1}x_{11}^{\prime },\cdots ,\lambda_{n}x_{1n}^{\prime },\cdots ,\lambda_{k}x_{k1}^{\prime },\cdots ,\lambda_{k}x_{kn}^{\prime }\right]$$

(23) $$Y=X^{\left[4\right]}W^{D}$$

where $W^{D}$ is the weights of the fully connected layer.

**Figure 10:**
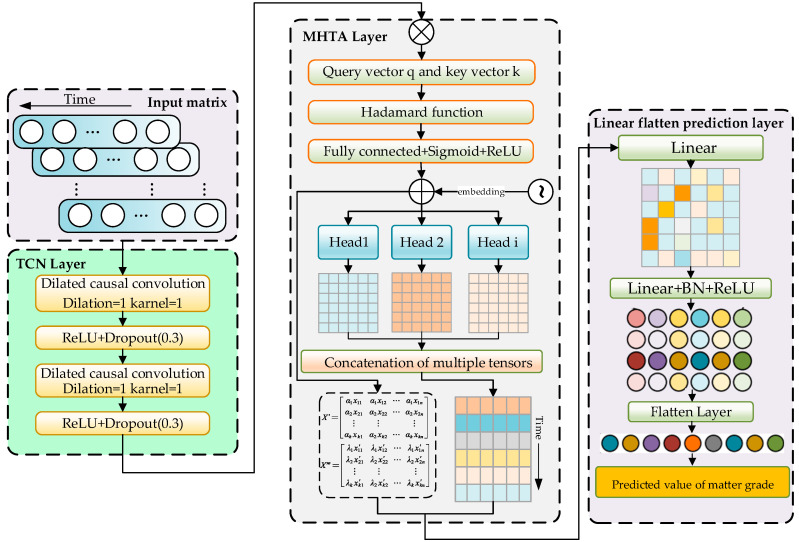
The process diagram for TCN-MHTA data processing.

The processing flowchart of the TCN-MHTA model is illustrated in Figure 10.

## 4. Experimental Results and Analysis

This paper verifies the effectiveness of the proposed method using a dataset related to copper smelting from a copper smelting plant in Yunnan. The experimental environment is configured as follows: i7-12700H CPU, NVIDIA GeForce RTX 3060, Python 3.7, and PyTorch 1.13.0. To validate the effectiveness of the model in the field of copper grade prediction and time series prediction. Firstly, the ablation experiments Model 1 to Model 3 were conducted on the Time2Vec and TCN-TMHA models, and Model 4 that did not consider temporal improvements. Then, four commonly used classical recurrent time series prediction models (SVM, LSTM, GRU, Transformer) are selected for comparative experiments. Finally, seven of the state-of-the-art (SOTA) models are chosen for comparison: GA-BP [6], GPR-DF [10], TimeNet [23], T2V-TF [24], PSO-TCN-Attention [25], BiTCN-MHTA [26], and BO-SVR [27]. An overview of the specific comparison methods is shown in Table 1.

### 4.1. Overview of the Dataset

The dataset includes the following:

(1) Parameters recorded manually: the contents of Cu, S, Fe, Si, and Ca in the input copper concentrate, the silicon and sulfur content of the flux, the amounts of fuel oil, coal, and slagging agents added, and the iron-silicon ratio in the slag,
(2) Data collected by industrial sensors: the amount of concentrate added, oxygen concentration, air blast volume, oxygen addition, molten pool temperature, burner height, burner back pressure, furnace chamber pressure, and the predicted copper grade label *Y*, as shown in Table 2.

The input variables *x* consists of the 19 process variables mentioned above time from 26 December 2022 to 25 December 2023, with time intervals varying between 55 and 70 min (the production cycle duration for each smelting operation is manually controlled). A total of 8747 data points were collected based on the above time intervals. After removing the downtime and maintenance data, there are 6991 valid data points. The data are split in a ratio of 7:2:1, resulting in a training set of 4893, a test set of 1398, and a validation set of 700 to assess prediction accuracy.

### 4.2. Parameter Settings and Hyperparameters’ Experiment

The training batch size for the prediction model in this paper is set to 32, with 1500 iterations. The parameter settings for each layer of the model are presented in Table 3. The Adam optimization algorithm is employed to optimize the other parameters of the prediction model, with a learning rate of 0.001.

Under multiple different experimental conditions, each model also made predictions on the copper smelting dataset. The results in four evaluation metrics clearly show that our proposed TCN-TMHA model achieved the best results among the various models, providing a more intuitive demonstration. After regular parameter settings, we conducted hyperparameter selection experiments to optimize the TCN model. Key hyperparameters affecting matte grade prediction, as shown in a related study [31], include batch size, neural network layers (neural layers), hidden units, and epochs. Using the Adam optimizer and cross-entropy loss function, we tested our TCN-TMHA model on two-month datasets (January and February 2023) to validate our findings.

**Figure 11:**
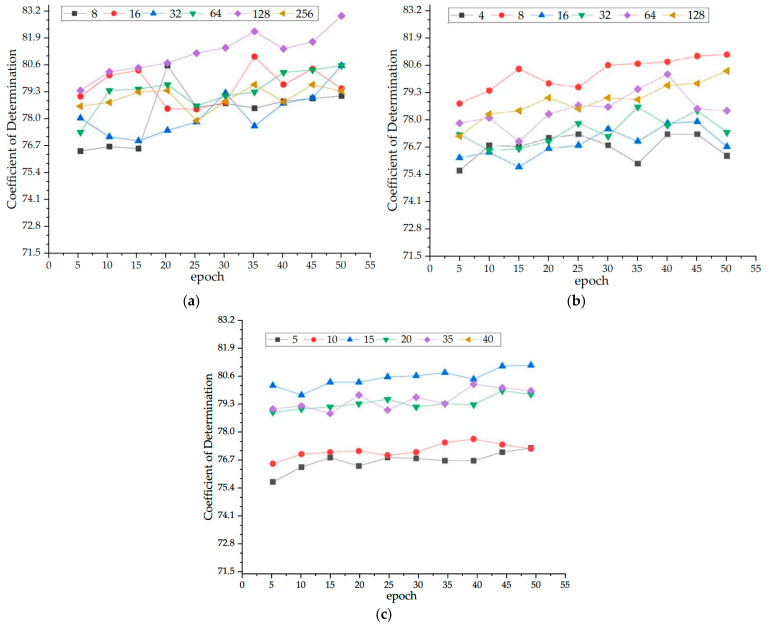
Experimental results of hyperparameter settings. (**a**) Hyperparameter experimental results of batch size. (**b**) Hyperparameter experimental results of neural layers. (**c**) Hyperparameter experimental results of hidden units.

As shown in Figure 11a, the model showed the best overall performance with a batch size of 128 on both datasets. Performance deteriorated at batch size 256, indicating overfitting. While a batch size of 32 showed good results in February, a batch size of 128 provided the best overall precision.

In the same way, Figure 11b,c indicate that four neural layers underperformed compared to eight due to non-convergence. Efficiency dropped past 8 layers due to overfitting. Hidden units of 20 provided the optimal detection results with smoother fluctuations, avoiding overfitting.

Our TCN-TMHA model achieved high accuracy starting at 20 epochs. To ensure precise predictions and align with other models, we set epochs to 50. Table 4 illustrates the results of LSTM, GRU, Transformer, SVR, and other models under different hyperparameter settings.

The evaluation metrics used in this paper consist of four indicators: Root Mean Square Error (*RMSE*), Mean Absolute Error (*MAE*), Mean Absolute Percentage Error (*MAPE*), and Coefficient of Determination (*R*^2^). The evaluation metrics can be found in Equations (24)–(27).

(24) $$RMSE=\sqrt{\frac{1}{n}\sum_{i-1}^{n}{\left(y_{i}-{\hat{y}}_{i}\right)}^{2}}$$

(25) $$R^{2}=1-\frac{\sum_{i=1}^{n}{\left(y_{i}-{\hat{y}}_{i}\right)}^{2}}{\sum_{i=1}^{n}{\left(y_{i}-{\bar{y}}_{i}\right)}^{2}}$$

(26) $$MAE=\frac{1}{n}\sum_{i=1}^{n}\left|y_{i}-{\hat{y}}_{i}\right|$$

(27) $$MAPE=\frac{1}{n}\sum_{i=1}^{n}\left|\frac{y_{i}-{\hat{y}}_{i}}{y_{i}}\right|$$

In the equations, ${\hat{y}}_{i}$ represents the predicted values, $y_{i}$ represents the true values, ${\bar{y}}_{i}$ denotes the mean, and n indicates the number of samples in the test set.

### 4.3. Ablation Experiment Setup and Results

To verify that the Time2Vec module, which characterizes temporal information in copper smelting, indeed affects the fluctuations in copper grade, this paper employs the Augmented Dickey–Fuller (ADF) test to examine the *p*-value and t-statistic of the input time series before and after the inclusion of the Model 1. Therefore, this paper chooses to use the ADF test to verify the performance of the Time2Vec module.

The *p* can be found in Equation (28):

(28) $$\Delta y_{t}=\alpha +\beta t+\gamma y_{t-1}+\sum_{i=1}^{p}\delta_{i}\Delta y_{t-i}+\varepsilon_{t}$$

where $\Delta y_{t}$ is the first difference in the time series, $\sum_{i=1}^{p}\delta_{i}\Delta y_{t-i}$ are the lag coefficients of the differenced series, and $\varepsilon_{t}$ is the error term. The *p*-value is derived from the t-statistic and represents the probability of observing the current t-statistic or a more extreme value, given that the null hypothesis (i.e., the time series is non-stationary, γ = 0) is true.

The statistic can be found in Equation (29):

(29) $$t=\frac{\hat{\gamma }}{SE(\hat{\gamma })}$$

where the t-statistic is the ratio of the estimated coefficient $\hat{\gamma }$ to its standard error $SE(\hat{\gamma })$, a larger t-statistic indicates that the time series is more likely to reject the null hypothesis of non-stationarity.

As shown in Table 5, nine sets of data containing temporal information were analyzed, including copper ore addition (*x*1), oxygen concentration (*x*2), fuel addition *(x*4), the iron-silicon ratio in slag (*x*12), furnace pressure (*x*15), molten pool temperature (*x*17), burner height (*x*18), and burner back pressure (*x*19). The elemental contents of copper, iron, silicon, and sulfur were manually recorded and do not exhibit temporal periodicity.

**Figure 12:**
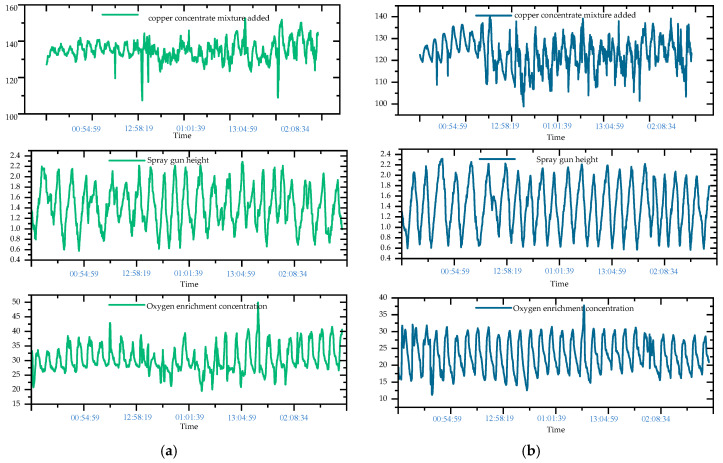
The trend of sensor process data over time in the database. (**a**) Example 1: data before being processed by the Time2Vec module. (**b**) Example 2: data after being processed by the Time2Vec module.

A specific visual comparison of data changes Model 1 and ours, using the data for addition amount, oxygen-rich concentration, and slag iron–silicon ratio as examples, shows that the *p*-value changes most significantly after the inclusion of Time2Vec, as illustrated in Figure 12.

**Figure 13:**
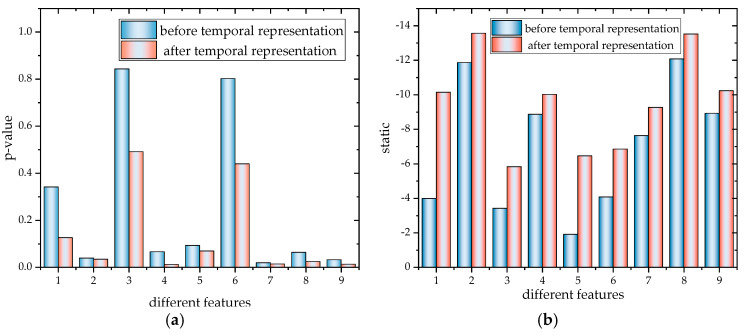
Changes in ADF values before and after characterization. (**a**) Example 1: *p*-value before being processed by the Time2Vec module. (**b**) Example 2: static before being processed by the Time2Vec module.

The data show that, for these three representative variables, the *p*-value changes from 0.3417 to 0.1256, from 0.8023 to 0.4391, and from 0.0192 to 0.0140, respectively. Additionally, the t-statistic values increase from −3.981 to −10.141, from −4.074 to −6.851, and from −7.629 to −9.269, indicating an improvement in stability. As an indicator for temporal stability and periodicity testing, the decrease in *p*-value indicates that the temporal periodicity of the data has been enhanced after integrating the periodic and linear modes of Time2Vec. Meanwhile, the increase in the statistics further supports the enhancement of the time series stationarity, indicating a stronger rejection of the non-stationarity hypothesis. These changes demonstrate that the Time2Vec module effectively improves the stationarity and periodicity of the time series, thereby stabilizing the copper smelting process and reducing fluctuations. The overall trend is shown in Figure 13.

To validate the effectiveness of each component in the proposed prediction model TCN-MHTA, ablation experiments resulting in three ablation models: Model 2, which removes the TMHA module; Model 3, which eliminates the temporal convolutional network (TCN) module; and Model 4, which only considers data changes without considering temporal periodicity and uses CNN-MHA for feature extraction. Through these ablation experiments, the results of the ablation experiments are shown in Table 6, and we can assess the contribution of each part to the overall performance of the model.

From the overall prediction results, it can be seen that the proposed model improves the prediction goodness-of-fit *R*^2^ by 8.233% compared to Model 2 (without TCN), while the prediction errors *RMSE*, *MAE*, and *MAPE* decrease by 7.015%, 18.620%, and 14.179%. Compared to Model 3 (without TMHA), the prediction goodness-of-fit *R*^2^ increases by 7.519%, and the prediction errors *RMSE*, *MAE*, and *MAPE* also decrease.

Particularly, it can be seen that, although the absolute errors for each value do not show significant improvement, the inclusion of feature extraction results in a notable reduction in *MAPE*, which is highly sensitive to large errors. This indicates that the prediction results are more accurate for high-frequency points, leading to a commendable improvement in the overall evaluation metric, *R*^2^. This indicates that the inclusion of TCN-TMHA can effectively enhance the model suitability and accuracy of copper content prediction.

**Figure 14:**
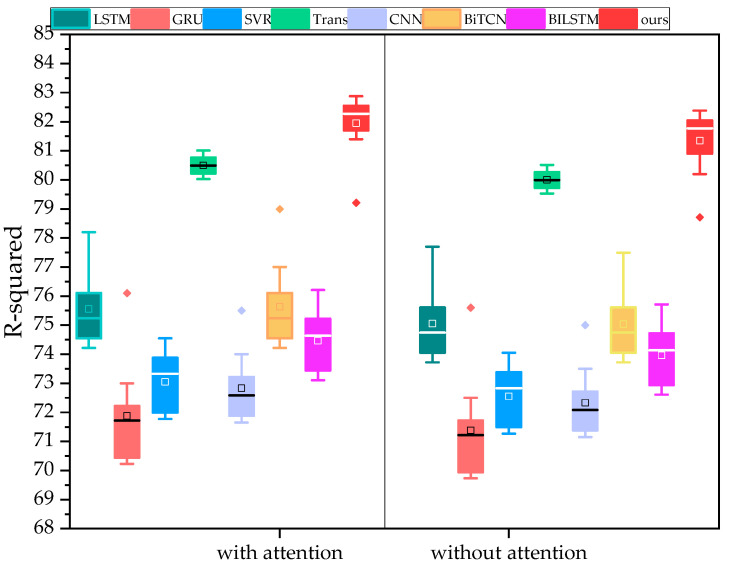
Comparison of model results before and after incorporating attention.

We also compared various baseline models before and after incorporating the attention mechanism under four evaluation metrics. For each baseline model, we conducted 10 experiments before and after adding the attention mechanism. The experimental results are shown in Figure 14.

Under the four evaluation metrics, our proposed TCN-based TCN-TMHA model achieved the best prediction accuracy results. (Note: *R*^2^ is better when larger, while *RMSE*, *MAPE*, and *MAE* are better when smaller. To maintain a consistent visual effect, the *y*-axis for the latter metrics was inverted).

Additionally, to discuss the effectiveness of the temporal periodic feature extraction module considered in this study, a comparison was performed between the proposed model and the CNN-MHA module that does not consider periodic features. The proposed model shows an improvement in prediction goodness-of-fit *R*^2^ of 20.206%, with prediction errors *RMSE*, *MAE*, and *MAPE* decreasing by 17.597%, 32.105%, and 35.694%, respectively. Our model shows a significant improvement compared to Model 4. This indicates that the use of the temporal model TCN-TMHA contributes greatly to the accuracy of the model, as further temporal modeling of time steps significantly enhances the performance compared to the single regression model CNN-MHA (Model 4). Comparing the accuracy before and after adding the attention mechanism in each baseline model, it is clear that the attention mechanism indeed enhances prediction accuracy.

In summary, the combination of TCN and TMHA in this study provides a greater enhancement to prediction performance, demonstrating the validity of the proposed model.

### 4.4. Analysis of Experimental Results

#### 4.4.1. Data Preprocessing Results

**Figure 15:**
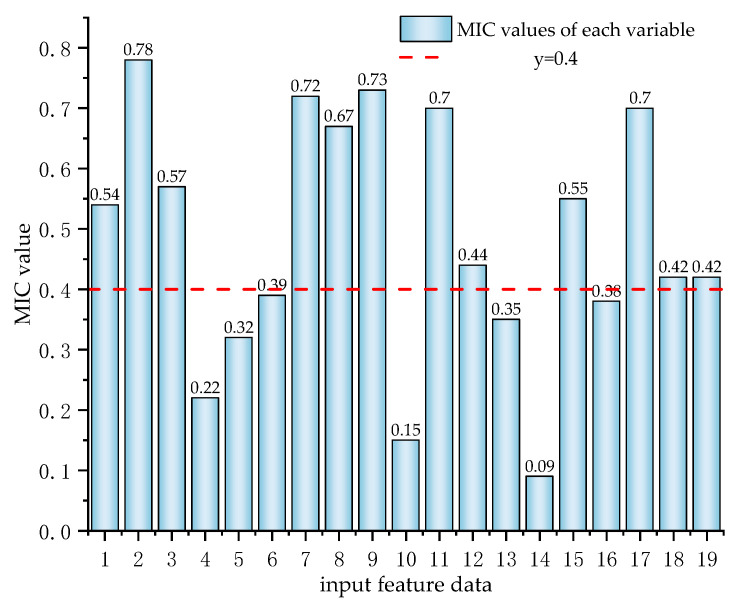
The MIC correlation of input variables.

Due to the low correlation characteristics among variables in the copper smelting process itself, we consider that, according to the definition of MIC and the reference, to better capture the relationships and improve the model’s predictive performance, we have utilized this MIC threshold to guide the selection of input variables. By focusing on variables with strong correlations (MIC > 0.4), we aim to include only those that have a meaningful impact on the output, thereby reducing noise and enhancing the model’s efficiency. The process of selecting input variables based on this MIC threshold is visually represented in Figure 15, which illustrates the variables retained for analysis and those discarded due to weak correlations. This approach not only aids in refining the model’s architecture but also contributes to a more robust and reliable prediction system in the context of copper smelting processes.

The variable correlations are shown in Figure 15. Ultimately, we selected the variables that are highly correlated with the copper grade as model inputs to reduce redundancy, including *x*1 (copper ore addition): 0.54; *x*2 (oxygen enrichment concentration): 0.78; *x*3 (fuel addition): 0.57; *x*7 (copper content): 0.72; *x*8 (iron content): 0.67; *x*9 (silicon content): 0.73; *x*11 (sulfur content): 0.7; *x*12 (iron–silicon ratio in the slag): 0.44; *x*15 (furnace pressure): 0.55; *x*17 (molten pool temperature): 0.70; *x*18 (burner height): 0.42; and *x*19 (burner back pressure): 0.42, totaling 12 process variables.

**Figure 16:**
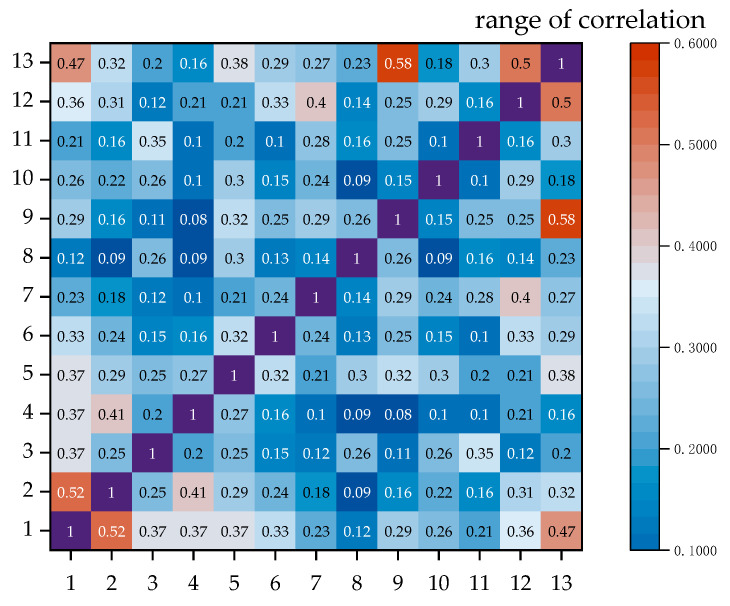
The correlation between the selected variables.

The variables that we discarded include fuel addition amount, slag-making agent silicon addition amount, blast volume, Ca content, and flux element content. These variables, such as flux and slag-making agents, are not added constantly, so they often do not affect the matte grade value. The main elements with high proportions in copper concentrate, namely, Cu, Fe, S, and Si, have all been selected. The Ca content is relatively low, which led to its exclusion during screening. As for the blast volume, it is customized and mainly adjusts the oxygen content of the smelting furnace by regulating the oxygen flow rate. The correlation of data after screening is shown in Figure 16.

#### 4.4.2. Time Series Feature Extraction Results Analysis

**Figure 17:**
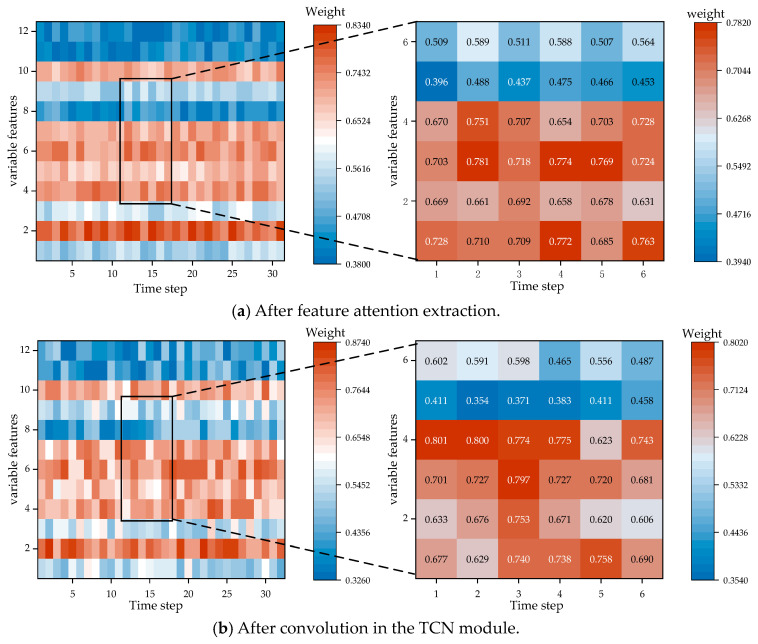

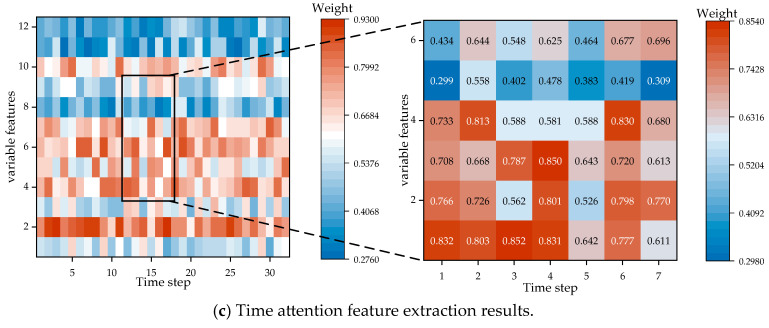
Attention weight heatmap.

In this section, we mainly explain the TMHA of the TCN-TMHA model to further validate the effectiveness. The discussion is based on the copper smelting process dataset. A heatmap visualization of the attention weight matrix is plotted, as shown in Figure 17.

The prediction task is to forecast the copper concentrate grade for the next production cycle time step based on historical data of 32 copper concentrate grades and 12 process variables, resulting in a matrix shape of 32 × 12, focusing on the results of the six time steps (one cycle) for the selected six groups of variables *x*7, *x*8, *x*9, *x*11, *x*12, and *x*15. Overall, the feature extraction module based on time attention further divides the time steps. Before extraction, the time step weights for almost every row of variables were almost consistent. After extraction, the module further differentiates the impact of different time steps on the results. Taking the data for *x*7 (copper content in copper ore) as an example, the color of the original feature extraction is almost entirely red, while the time attention module deepens the weights of 1st to the 3rd time steps and reduces the weights of time steps 4 and 6, indicating that the first 30 min before smelting have a greater impact on the results, thereby improving prediction accuracy.

**Figure 18:**
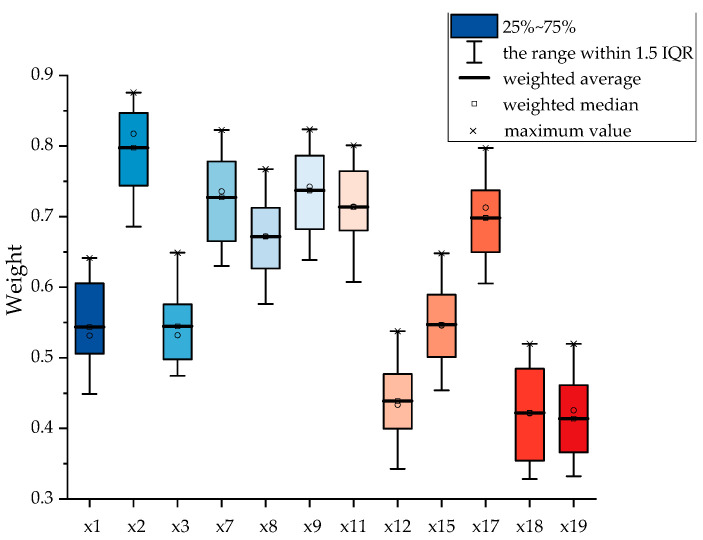
Box plot of feature weights extracted by attention.

From the interpretive perspective of Figure 18, it is evident that the features from the 2nd to the 5th time step and the weights from the first four time steps are given more importance. This aligns closely with the high correlation results obtained from the MIC analysis in Section 3.2. Specifically, the amount of copper concentrate added, the Cu content in the ore, molten pool temperature, oxygen concentration, and burner height significantly determine the final copper concentrate production. This conclusion is largely consistent with the findings in reference [28].

**Figure 19:**
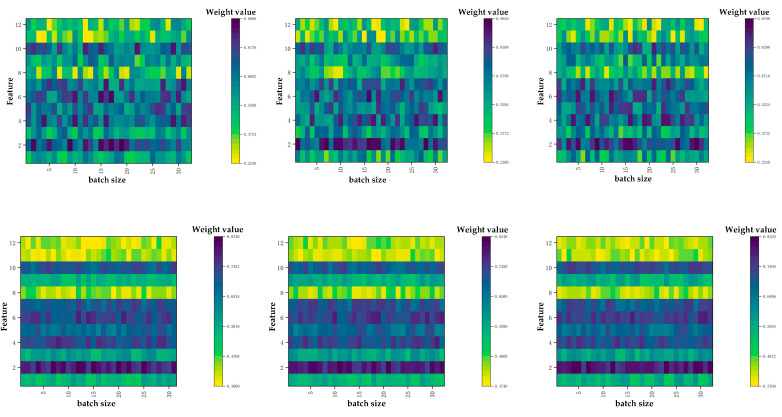
The trend of sensor process data over time in the dataset.

We have incorporated the results of multiple experiments from Figure 19 into the drawing for a detailed illustration of the time patterns we identified and their actual impact, and our TCN-TMHA module is capable of extracting features quite clearly. It can be observed that the first few time steps (highlighted in green) have a relatively significant influence, while the later time steps (highlighted in yellow) have a much smaller impact on the copper smelting process results. This distinction highlights the varying influence of different time steps within a cycle, which is indeed of great significance in actual smelting operations. It allows on-site personnel to focus more closely on these critical periods, thereby optimizing the production process.

#### 4.4.3. Comparative Analysis of Copper Matte Grade Prediction Results

To verify the accuracy of the model in predicting matte grade, three relatively new prediction models were selected for comparative experimental analysis using the dataset from this study: GA-BP [6], GR-GRM (Gauss process regression–dynamic regression modeling) [17], and BO-SVR [25]. The results are shown in Table 7.

**Figure 20:**
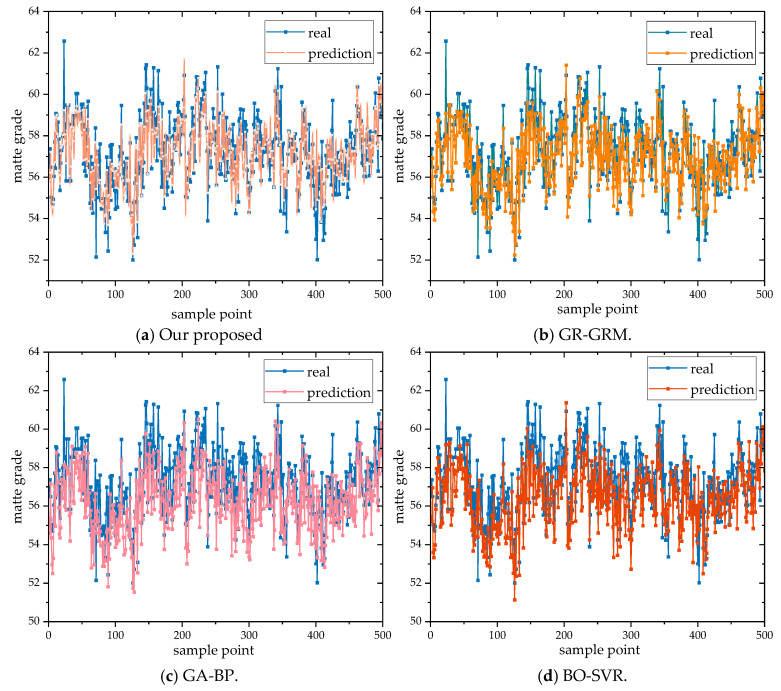
Comparison chart of prediction models for matte grade.

According to Table 7, the point prediction accuracy of our model is the highest, with *RMSE*, *MAE*, and *MAPE* values of 0.782, 0.590, and 0.692, respectively, and an *R*^2^ of 0.815. When applied to our copper smelting dataset, our model significantly outperforms models obtained through BO-SVR, GA-BP combined with chemical analysis, and GR-GRM time series analysis, showing improvements in *R*^2^ and reductions in *RMSE*, *MAE*, and *MAPE*. Specifically, our model achieved *R*^2^ improvements of 11.675%, 17.604%, and 2.13%, respectively, with corresponding decreases in error metrics. This demonstrates that our model provides more accurate predictions of copper grade and highlights the effectiveness of time series analysis on model performance. As illustrated in Figure 20, our model exhibits overall lower errors on the test set and outperforms GR-GRM, GA-BP, and BO-SVR models.

**Figure 21:**
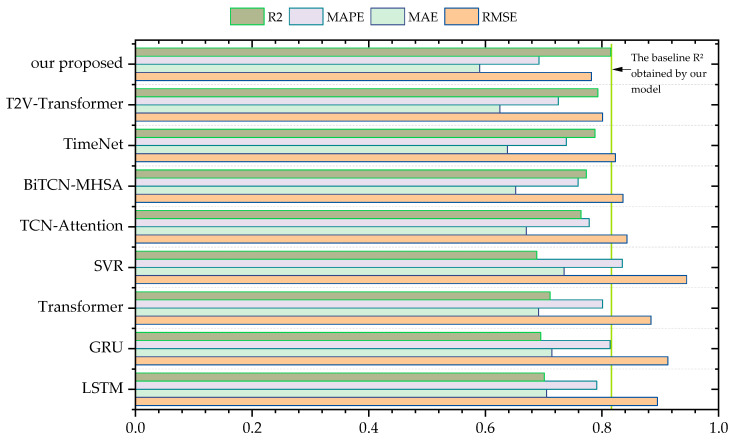
Visualization of multi-model comparison results.

To verify the effectiveness of the proposed model, we selected four commonly used time series prediction models: LSTM, GRU, Transformer, and SVR. Additionally, we compared the results obtained from these four models with those from four advanced time series prediction models: TimeNet, PSO-TCN-Attention, Time2Vec-Transformer, and BiTCN-MHTA. The comparison was conducted using the dataset employed in this study. The results are visually presented and analyzed in Figure 21, which highlights the performance metrics and key outcomes of each model, providing a comprehensive understanding of their predictive capabilities and accuracy.

The comparative analysis not only highlights the strengths and weaknesses of each model but also provides valuable insights into their respective application domains. For instance, models like LSTM and GRU, known for their ability to capture temporal dependencies in sequential data, demonstrated robust performance in datasets with clear time-correlation patterns. On the other hand, the Transformer model, leveraging its self-attention mechanism, excelled in tasks requiring a holistic view of the temporal sequence, making it suitable for datasets with complex nonlinear dependencies.

**Figure 22:**
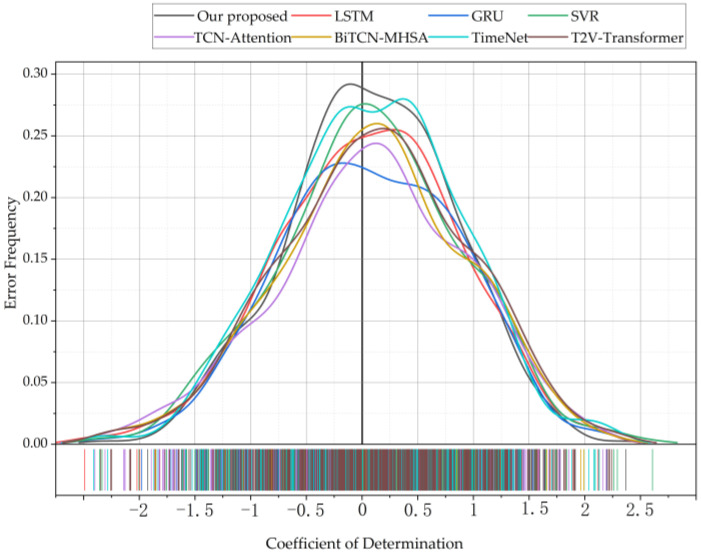
Results of each model under statistical confidence.

The prediction error distribution curves of different models are illustrated in Figure 22. As seen in this figure, the proposed model in this paper exhibits the most concentrated distribution of prediction errors around the zero value on the test set. Additionally, its distribution curve is narrower compared to those of other models, indicating that the proposed model has smaller overall errors and better prediction performance on the test set.

The results of these experiments using our copper smelting dataset are shown in Table 8, and it is evident that our proposed model achieved the best experimental results, outperforming all other benchmark and advanced models included in the study. Specifically, our model’s ability to dynamically adjust its learning parameters and capture intricate temporal patterns sets it apart from traditional and contemporary alternatives.

In our copper smelting dataset evaluation, our proposed model demonstrated significant improvements over several models: compared to LSTM, *R*^2^ increased by 16.262%; compared to GRU, *R*^2^ increased by 17.266%; compared to Transformer, *R*^2^ increased by 14.627%, with *RMSE*, *MAE*, and *MAPE* decreasing by 11.538%, 14.616%, and 13.607%, and compared to SVM, *R*^2^ increased by 18.459%, exhibiting the largest enhancements in *RMSE*, *MAE*, and *MAPE*, with decreases of 17.248%, 19.727%, and 17.125%.

**Figure 23:**
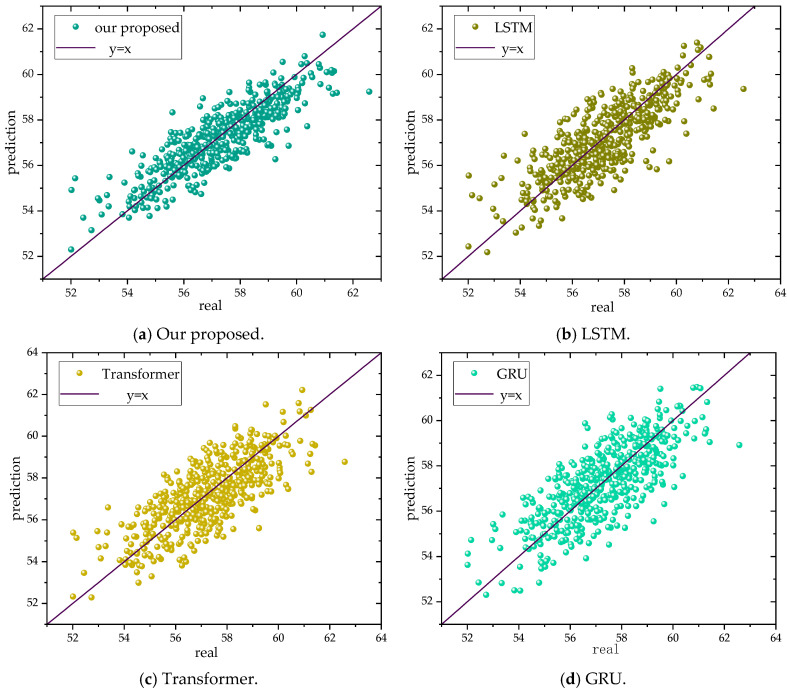
Comparison with common time series models.

It is evident that our proposed model shows improvements over the baseline models, allowing for more accurate predictions of copper grade. Moreover, the results obtained from these baseline models are nearly consistent, with minimal differences. To illustrate the advantages of our model in predicting absolute errors, we used a linear function graph of *y* = *x* to visualize our performance, as shown in Figure 23. By extracting temporal signals, our model demonstrates a significant improvement in prediction capabilities for time series data. Specifically, the model excels in handling frequently occurring data points, substantially enhancing the prediction accuracy for these critical time points.

We compared our model with various SOTA models, with the results summarized in Table 9. Compared to the TCN-Attention model, our proposed model achieved an *R*^2^ improvement of 6.675%, with *RMSE*, *MAE*, and *MAPE* decreasing by 7.236%, 11.940%, and 11.053%, respectively, showing the most significant enhancements. In comparison to the T2V-Transformer model, our model achieved an *R*^2^ increase of 2.774%, with *RMSE*, *MAE*, and *MAPE* decreasing by 2.372%, 5.600%, and 4.551%, respectively, indicating the smallest improvements. In contrast, our model showed *R*^2^ improvements of 5.433% and 3.426% when compared to the BiTCN-MHSA and TimeNet models, respectively.

As shown in Table 9, our proposed model has a 95% data error interval of [−1.693, 1.826], while the error intervals for the other three models BiTCN-MHTA, T2V-TF, and TimeNet are [−1.105, 2.727], [−1.124, 3.139], and [−1.456, 2.070], respectively.

It is evident that each SOTA model has its advantages and performs better than the baseline models. Specifically, TCN-Attention and BiTCN-MHSA are recent models that employ similar architectures of dilated convolution combined with attention mechanisms, and they have demonstrated strong predictive performance. The advantage of our model compared to these two lies in the integration of the Time2Vec temporal representation module, which enhances the stability of the input sequences, resulting in better prediction outcomes.

**Figure 24:**
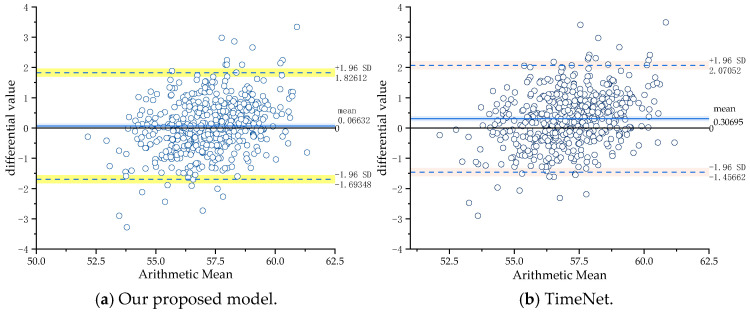

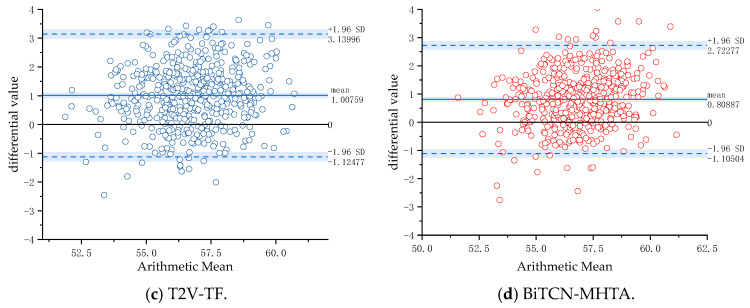
Time series prediction SOTA model comparison experiment.

To visually illustrate the advantages of our model compared to SOTA models, we employed a visualization method using a 95% confidence interval, as shown in Figure 24.

Conclusively, our model has an average error of 0.063, which represents a significant accuracy improvement compared to the other models with average errors of 0.808, 1.007, and 0.306. This allows for a more accurate prediction of copper grade. The prediction accuracy of the proposed method is the highest, which proves the effectiveness of the proposed method.

## 5. Conclusions

In this paper, we propose a prediction model based on Time2Vec and TCN-TMHA to address the issue of weak temporal representation in copper smelting production monitoring data and to enhance the ability of existing models to extract periodic features. These improvements enable time series prediction of the matte grade during the copper smelting process.

(1) Based on the proposed model, our research results show that the Time2Vec module successfully extracted the periodic information from the copper smelting process by using the TCN-TMHA module to assign weights to the input features and time steps, and we significantly enhanced the impact of key temporal information.
(2) Compared to traditional regression models and advanced models, the proposed model demonstrates significant improvements in predicting copper content, with the *R*^2^ increasing by 11.675%, 17.604%, and 2.130%. The combination architecture of TCN-TMHA and Time2Vec embedding effectively captures the temporal features within the dataset, leading to improved prediction accuracy.

In the future, exploring models that can control copper content will be an important research direction. This not only helps to optimize the production process but also enhances the quality and consistency of products. Moreover, a deeper investigation into the relationship between process variables and copper content will help us better understand how these variables influence the quality of the final product. Through precise control of process variables, we can more effectively manage copper content, thereby ensuring quality while reducing costs.

## Figures and Tables

**Table 1:** The experimental method design of this paper.

Experimental Method		Description of the Comparative Models		
Ablation experiment	Our proposed	Time2Vec	TMHA	TCN
	Model 1	\	TMHA	TCN
	Model 2	Time2Vec	\	TCN
	Model 3	Time2Vec	TMHA	\
	Model 4	Time2Vec	MHA	CNN
Comparison experiment of classical models	SVM	Introducing kernel functions to handle nonlinear capabilities		
	LSTM	Solving the gradient vanishing and exploding problems when learning long-term dependencies in sequences		
	GRU	Making the structure relatively smaller and training speed faster		
	Transformer	Using attention mechanisms to capture long-term dependencies		
Comparison experiment with SOTA models	GA-BP [6]	Implementation of copper grade prediction based on regression models		
	TimeNet [26]	A prediction model that automatically captures complex and nonlinear temporal information using Time2Vec-attention and CNN-BiGRU		
	GPR-DF [10]	A prediction framework for copper grade using Gaussian regression models and dynamic features		
	T2V-TF [27]	An adaptive learnable prediction model based on Time2Vec and Transformer		
	PSO-TCN-Attention [28]	Focus on the relationship between key time steps and parameters to make weight acquisition more accurate and faster		
	BiTCN-MHTA [29]	Improving the temporal prediction model of the TCN model using the cross-entropy loss function		
	BO-SVR [30]	Using machine learning methods to predict the copper content in slag and the copper grade		

**Table 2:** Relevant variables for matte grade prediction model.

Variables	Title 2	Title 3
*x*1	Copper concentrate mixture added	Weight of copper concentrate in furnace feed per hour (t/h)
*x*2	Oxygen enrichment concentration	Ratio of oxygen volume to total gas volume (%)
*x*3	Coal-to-material ratio	Weight of coal added to copper concentrate (%)
*x*4	Amount of fuel added	Weight of fuel in furnace feed per hour (t/h)
*x*5	Silicon additive for slag	Weight of copper concentrate in furnace feed per hour (t/h)
*x*6	Blowing volume	Air amount supplied to furnace per hour (t/h)
*x*(7–11)	Content of copper concentrate	Elemental content of ore in copper concentrate in furnace feed (%)
*x*12	Iron–silicon ratio in slag	Ratio detected from discharged slag (%)
*x*(13,14)	Elemental content in flux	Elemental content in flux used for combustion (%)
*x*15	Furnace pressure	Internal pressure in smelting furnace (kPa)
*x*16	Amount of oxygen added	Amount of oxygen supplied to furnace per hour (t/h)
*x*17	Molten pool temperature	Fuel molten pool temperature in smelting furnace (°C)
*x*18	Spray gun height	Height of gun above molten pool (m)
*x*19	Spray gun back pressure	Internal pressure of molten pool (kPa)
*Y*	Matte grade	Percentage of copper amount after smelting to total amount (%)

**Table 3:** Model parameters of the proposed TCN-MHTA.

Model Construction	Parameter Name	Value	Parameter Name	Value
Feature Extraction	Number of Attention Heads	3	Matrix Dimensions	3 × 3
TCN Layer	Number of Filters	20/16	Padding	Casual
	Filter Size *k*	2	Number of Stacked Layers	1
	Dilation Factor *d*	[1, 2, 4, 8]	Number of Residual Blocks	2
	Dropout	0.2	Activation Function	Relu
Attention Extraction	Number of Attention Heads	3	Matrix Dimensions	3 × 3
Fully Connected	Number of Neurons	1	Activation Function	Tahn

**Table 4:** Baseline model parameter settings for comparative experiments.

	TCN	LSTM	GRU	SVR	Transformer
Batch size	128	128	64	32	128
neural layers	8	8	4	2	4
hidden units	20	15	10	10	20
gamma	0.9	0.9	\	0.9	0.9
epochs	50	50	50	50	50
lr	0.001	0.001	0.001	0.001	0.001
loss_function	*MSE*	*MSE*	*MSE*	*MSE*	*MSE*
optimization	Adam	Adam	Adam	Adam	Adam

The baseline model does not undergo hyperparameter tuning, but only illustrates the comparability in usability with the models presented in this paper.

**Table 5:** ADF changes in data before and after Time2Vec representation.

Variables	Without Time2Vec (Model 1)		With Time2Vec Module	
ADF Test Value	*p*-Value	Statistic	*p*-Value	Statistic
copper ore addition (*x*1)	0.3417	−3.981	0.1256	−10.141
oxygen concentration (*x*2)	0.0393	−11.863	0.0341	−13.565
fuel addition (*x*4)	0.8429	−3.417	0.4911	−5.833
silicon ratio in slag (*x*12)	0.0661	−8.868	0.0121	−10.024
furnace pressure (*x*15)	0.0928	−1.914	0.0695	−6.456
molten pool temperature (*x*17)	0.8023	−4.074	0.4391	−6.851
burner height (*x*18)	0.0192	−7.629	0.0140	−9.269
burner back pressure (*x*19)	0.0637	−12.079	0.0242	−13.523
matte grade (*Y*)	0.0321	−8.926	0.0131	−10.237

Since the preprocessing module based on MIC has already deleted some variables, and the copper concentrate content portion does not have a temporal sequence, it has not been included in this table.

**Table 6:** Baseline model parameter settings for comparative experiments.

Method	*RMSE*	*MAE*	*MAPE*	*R* ^2^
Model 2	0.841	0.725	0.804	0.753
Model 3	0.803	0.698	0.848	0.758
Model 4	0.949	0.869	1.073	0.678
Our proposed	0.782	0.590	0.690	0.815

**Table 7:** Baseline model parameter settings for comparative experiments.

	*RMSE*	*MAE*	*MAPE*	*R* ^2^
BO-SVR	0.949	0.769	0.825	0.728
GA-BP	1.046	0.852	0.839	0.693
GR-GRM	0.903	0.698	0.789	0.798
Our proposed	0.782	0.590	0.692	0.815

**Table 8:** Results of the time series model comparison experiments.

	*RMSE*	*MAE*	*MAPE*	*R* ^2^
LSTM	0.895	0.705	0.791	0.701
GRU	0.913	0.714	0.814	0.695
Transformer	0.884	0.691	0.801	0.711
SVR	0.945	0.735	0.835	0.688
TCN-Attention	0.843	0.670	0.778	0.764
BiTCN-MHSA	0.836	0.652	0.759	0.773
TimeNet	0.823	0.638	0.739	0.788
T2V-Transformer	0.801	0.625	0.725	0.793
Our proposed	0.782	0.590	0.692	0.815

**Table 9:** 95% confidence interval error precision and range.

	*Error Mean*	*95% Upper*	*95% Lower*	*R* ^2^
BiTCN-MHTA	0.808	2.727	1.105	0.728
T2V-TF	1.007	3.139	1.124	0.693
TimeNet	0.306	2.070	1.456	0.798
Our proposed	0.063	1.826	1.693	0.815

## Data Availability

Since the data are part of the ongoing research, the dataset provided in this paper is not easy to obtain.
